# Interaction of CETP rs708272 Polymorphism on Trans Fatty Acid Intake and Glucose Metabolism Markers

**DOI:** 10.3390/nu16213683

**Published:** 2024-10-29

**Authors:** Edgar J. Mendivil, Gerardo Barcenas-Rivera, Omar Ramos-Lopez, Cesar Hernández-Guerrero, Ingrid Rivera-Iñiguez, Yolanda E. Pérez-Beltrán

**Affiliations:** 1 Health Department, Universidad Iberoamericana, Mexico City 01219, Mexico; 2 Master in Applied Nutriology Program, Universidad Iberoamericana, Mexico City 01219, Mexico; 3 Medicine & Psychology Faculty, Autonomous University of Baja California, Tijuana 22390, Mexico; 4 UCSD Center for Healthy Eating and Activity Research (CHEAR), School of Medicine, University of California, San Diego, CA 92037, USA; 5 Laboratorio Nacional de Investigación para la Inocuidad Alimentaria (LANIIA)-Unidad Nayarit, Universidad Autónoma de Nayarit, Tepic 63173, Mexico

**Keywords:** dietary fats, trans fatty acids, *CETP* rs708272 polymorphism, HOMA-IR, precision nutrition

## Abstract

Dietary fats influence gene expression and several metabolic pathways. Therefore, it is crucial to study the role of personal genotypes in the interaction between fat consumption and cardiometabolic markers. This research aimed to determine the interaction of the rs708272 polymorphism of *CETP* and the fatty acid intake with changes in the HOMA-IR in adults living with overweight or obesity. The current study was a secondary analysis of an 8-week controlled clinical trial. The final sample for this analysis comprised 78 Mexican adults with the Cholesteryl Ester Transfer Protein (*CETP)* rs708272 polymorphism who followed a dietary intervention. Using an interaction analysis, we evaluated the fatty acid intake and the genotypes of rs708272, with changes in blood glucose, insulin, and the HOMA-IR from baseline to endpoint. Our findings suggest a significant interaction between the trans fatty acid intake and the GG genotype with changes in glucose (*p* = 0.024), insulin (*p* = 0.004), and the HOMA-IR (*p* = 0.002). The higher the consumption of trans fatty acids, the less these markers of glucose metabolism were reduced. carriers of the GG genotype may benefit from limiting dietary trans fatty acid intake, as there was no reduction in plasma glucose and insulin despite a hypocaloric dietary intervention in adults with overweight and obesity.

## 1. Introduction

Diabetes is a global public health issue characterized by elevated blood glucose levels, and, like obesity, it has a multifactorial origin [1]. These elevated blood glucose levels result from insulin deficiency, insulin resistance, or both. The most common type of diabetes worldwide is type 2 diabetes, accounting for over 90% of cases. This type of diabetes is characterized by insulin resistance, which leads to increased insulin production and, eventually, atrophy in pancreatic beta cells, reducing insulin secretion [2]. The International Diabetes Federation (IDF) estimated that in 2021, approximately 537 million people aged 20–79 were living with diabetes, representing 10.5% of the global population. Of these, around 6.7 million deaths were attributed to this disease [3]. By 2030, it is expected that this number will exceed 643 million people living with diabetes, with projections indicating that by 2045, one in eight adults worldwide will have diabetes [1,4]. The Homeostasis Model Assessment of Insulin Resistance (HOMA-IR) is a method widely used in clinical practice for predicting insulin resistance, as it is a relatively quick and practical approach. The HOMA-IR can be calculated as fasting insulin (μU/dL) * fasting glucose (mmol/L)/22.5 [5].

Precision nutrition focuses on providing holistic care in a most personalized way, including the exposome, traditional aspects such as sociocultural practices, lifestyle, and dietary preferences, as well as genetic factors (nutrigenetics), microbiota composition, metabolites (metabolomics), among others, to prevent chronic diseases and improve people’s quality of life [6]. Nutrigenetics, as part of precision nutrition, studies the role of genetic variants in achieving more precise nutritional care, leading to better outcomes than a one-size-fits-all approach [7]. Identifying polymorphisms can determine genetic markers that may confer risk or protection for certain conditions, allowing more successful nutritional treatment. Also, individual responses to dietary counseling can be predicted by analyzing the interaction between the genetic makeup and environmental factors, facilitating the prescription of personalized nutritional strategies.

The *CETP* codifies for an eponymous protein that is a plasma glycoprotein belonging to the lipid transfer protein (LTP) family, which facilitates the exchange of triglycerides for cholesterol esters from HDL to apolipoprotein B-containing lipoproteins, thereby reducing HDL cholesterol (HDL-C) concentration [8]. *CETP* is localized on chromosome 16q13, and its polymorphisms influence protein activity and plasma lipid profiles, affecting the development and progression of coronary artery disease (CAD). Three major single nucleotide polymorphisms (SNPs) of *CETP* have been extensively studied and associated with CAD risk. These SNPs are rs708272, rs5882, and rs180075. *CETP* rs708272 (*TaqI* B) involves the substitution of G for A at nucleotide 277 (G277A) and is associated with elevated HDL-C levels due to decreased *CETP* expression. However, various studies show contrasting results across ethnic groups regarding which allele is the risk factor for metabolic alterations [9,10,11].

*Trans*-fatty acids (TFA) are fatty acids that contain at least one unsaturation (double bond) in their carbon chain, adopting a trans configuration [12]. Although they can be produced through food processing as well as in cellular metabolism, they have gained significant importance for health because they are commonly found as additives in ultra-processed foods [13]. TFA intake has been associated with an increased risk of developing cardiovascular diseases. The main proposed mechanisms of action include their impact on blood lipid levels, their potential behavior as free radicals, and their triggering of proinflammatory processes. For this reason, the consumption of TFA could also be associated with developing insulin resistance [14,15,16]. In this study, we focused on examining the interaction of the rs708272 polymorphism on the effect of TFA consumption on the Homeostatic Model Assessment of Insulin Resistance (HOMA-IR) HOMA-IR indicators in adults with overweight and obesity following a hypocaloric diet for weight reduction.

## 2. Materials and Methods

### 2.1. Study Population

The present research is an ancillary study of the randomized controlled trial conducted by Pérez-Beltrán et al., [17] (ClinicalTrials.gov: NCT05210023), in which seventy-eight individuals of Mexican descent with overweight or obesity (BMI 25 ≥ 40 kg/m^2^) followed a control weight diet during eight weeks. The participants resided in the metropolitan area of Guadalajara, Jalisco, in western Mexico. They were between 18 and 50 years old, with a waist circumference >94 cm for men and >80 cm for women. The study excluded individuals with certain medical conditions, including cardiovascular disease, mental illness, diabetes, and gastrointestinal disorders, among others. Participants were recruited and assessed at the University of Guadalajara and Jesuit University of Guadalajara (ITESO), all providing informed consent. This research adhered to the Declaration of Helsinki and was approved and registered under the 0001DRC number by the ITESO ethics committee.

### 2.2. Genotyping

Before starting the intervention, blood samples were collected from participants for genotyping. DNA was extracted from leukocytes using a DNA extraction kit (High Pure PCR Template Preparation kit, Roche Diagnostics, Mannheim, Germany), following the manufacturer’s instructions. The quality and quantity of the genomic DNA were assessed using a microvolume spectrophotometer (Multiskan SkyHigh, ThermoFisher Scientific, Waltham, MA, USA). DNA samples were genotyped using TaqMan SNP genotyping assays (CETP rs708272, assay ID 4351379 C___9615318_10, Context Sequence [VIC/FAM] CTGAGACCCAGAATCACTGGGGTTC[A/G]AGTTAGGGTTCAGATCTGAGCCAGG, Thermo Fisher Scientific: https://www.thermofisher.com accessed on 12 September 2021) in a 96-well format, and analyzed with a Roche LightCycler 96 system (Roche, Mannheim, Germany). The PCR protocol included an initial denaturation at 95 °C for 10 min, followed by 40 cycles of annealing/extension at 60 °C for 60 s.

### 2.3. Dietary Intake

According to WHO recommendations, the participants followed a healthy, low-calorie diet for weight control [18]. The energy prescription for participants was calculated based on 25–30 kcal/kg of ideal weight, with a macronutrient distribution of 50% carbohydrates, 25% protein, and 25% fat (including 6–11% polyunsaturated fatty acids (PUFAs), 15% monounsaturated fatty acids (MUFAs), and >10% saturated fatty acids. Four standardized weekly diet options, ranging from 1000 to 2200 kcal, were created using ESHA’s Food Processor^®^ Nutrition Analysis software (version 11.11, Rockford, IL, USA) and included five mealtimes (breakfast, morning snack, lunch, evening snack, and dinner).

Participants recorded their daily intake of macronutrients and fatty acids using the five-step 24-h recall for baseline data and a 3-day diet record, which included two weekdays and one weekend day for final data. The participants were instructed on how to complete the records, and trained dietitians coded the food records using ESHA’s Food Processor^®^ software (version 11.11, Rockford, IL, USA). Nutrient intakes were averaged over the three days, and adherence to the diet was evaluated through the percentage of adequacy: %AD = [consumed daily intake (g)/recommended daily intake (g)] × 100.

### 2.4. Anthropometric and Biochemical Measurements

Trained dietitians collected anthropometric measurements, including body weight (kg), waist circumference (WC, cm), and hip circumference (HC, cm), using conventional validated methods [19]. Body Mass Index (BMI) was calculated as weight (kg) divided by height (m^2^). Total body fat mass (TBF, kg) and skeletal muscle mass (MuscleM, kg) were measured using electrical bioimpedance (InBody 120, Segmental Multi-frequency Body DSM-BIA, Seoul, Republic of Korea).

Peripheral blood samples were collected after a 10-h overnight fast and immediately centrifuged at 3500 rpm to obtain serum. Biochemical variables, including glucose (mg/dL), total cholesterol (TC, mg/dL), triglycerides (TG, mg/dL), high-density lipoprotein cholesterol (HDL-C, mg/dL), and very low-density lipoprotein cholesterol (VLDL-C, mg/dL), were analyzed using a dry chemistry analyzer (Vitros 250 Analyzer, Ortho-Clinical Diagnostics, Johnson & Johnson Services, Inc., Rochester, NY, USA). Low-density lipoprotein cholesterol (LDL-C, mg/dL) was calculated using the Friedewald formula. Serum insulin levels (µUI/mL) were measured using a LIASON^®^ immunoassay kit (Insulin ref: 310360 Diasorin Liaison^®^, Milan, Italy). The homeostatic model assessment of insulin resistance (HOMA-IR) was calculated from fasting blood plasma glucose and serum insulin values [20], with a HOMA-IR value >2.5 indicating insulin resistance. Both baseline and final measurements were considered for this study.

### 2.5. Statistical Analyses

Data are expressed as means ± standard deviations. The Kolmogorov–Smirnov test was used to analyze the normality of the variables, and the Levene test was used to assess the equality of variances. Paired and unpaired Student’s *t*-tests were applied. Data analysis was performed using IBM SPSS version 26 (Statistical Package for the Social Sciences, Chicago, IL, USA), with a significance level set at *p* < 0.05. The *p*-value for differences in quantitative variables was determined using the Wilcoxon test. An interaction analysis was conducted using multivariate linear regression models, adjusted for age, sex, total calories, and body mass index (BMI), and corrected by multiple comparisons using the Bonferroni test. A 95% confidence interval and a significant *p*-value of < 0.05 were considered. The statistical analyses were performed using IBM SPSS Statistics version 29.0.2.0 and StataCorp 2023 Stata statistical software version 18 (StataCorp LLC., College Station, TX, USA; www.stata.com).

The genetic variation in the population was tested for Hardy–Weinberg equilibrium (HWE) using a χ^2^ test.

## 3. Results

### 3.1. Demographic Characteristics of the Study Population

Table 1 shows the study participants’ sociodemographic characteristics and their respective *CETP* rs708272 genotypes. The mean age of the analyzed population (n = 78) was 34.6 ± 8.1 years; 38% (n = 38) of the individuals were men, and 52% (n = 40) were women.

Genotype frequency was not significantly different from that predicted by the Hardy–Weinberg equilibrium. Twenty-four individuals (31%) possessed the GG genotype (B1B1), and given the number of individuals carrying the AA genotype (n = 14), they were grouped with individuals with the GA genotype (n = 40), thus forming a group comprising 54 (69%) individuals carrying at least an A allele (B2). There were no statistically significant differences between the proportion of women or men carrying the B1B1 or B2 genotypes.

### 3.2. Dietary Intake

The nutritional intake of the participants is shown in Table 2. A significant difference was observed when comparing the baseline and final (after eight weeks) consumption of calories (∆ = −392.32 ± 833.04, *p* < 0.001), carbohydrates (∆ = −73.83 ± 166.22, *p* < 0.001), and total lipids (∆ = −19.84 ± 45.18, *p* < 0.001), among which a significant decrease was reported only in the intake of saturated fatty acids (SFA) and cholesterol (∆ = −6.86 ± 15.59, *p* < 0.001 and −59.674 ± 216.99, *p* = 0.020, respectively). Meanwhile, the consumption of trans fatty acids (∆ = −0.106 ± 0.536, *p* = 0.733), polyunsaturated fatty acids (PUFA) (∆ = −3.212 ± 12.49, *p* = 0.149), and monounsaturated fatty acids (MUFA) (∆ = −4.17 ± 18.31, *p* = 0.053) remained unchanged during the intervention, as did the amount of protein consumed by the participants (∆ = −1.92 ± 54.05, *p* = 0.941).

### 3.3. Anthropometric, Body Composition, and Biochemical Parameters

The results obtained from the anthropometric evaluation, body composition, and biochemical parameters are reported in Table 3. Favorably, a statistically significant change (*p* < 0.05) was observed in most of the evaluated parameters when compared to the baseline data.

According to the BMI classification [21], the population’s mean was categorized as Obesity 1 (30.60 ± 4.08) at the beginning of the intervention. However, a significant reduction in the body weight of the participants was reported (∆ = −3.48 ± 3.06, *p* < 0.001), resulting in final BMI values classifying them as overweight (29.34 ± 4.08, ∆ = −1.26 ± 1.02, *p* < 0.001).

The baseline average waist circumference (WC: 94.96 ± 12.14 cm) and hip circumference (HP: 110.30 ± 7.91 cm) were also above the recognized healthy parameters (WC men: <94 cm, women <80 cm). However, after the 8-week intervention, a significant reduction was observed in both parameters (WC: 90.85 ± 11.37 cm, ∆ = −4.10 ± 4.46, *p* < 0.001; HP: 107.31 ± 7.87 cm, ∆ = −2.98 ± 2.81, *p* < 0.001).

Total body fat mass (TBFM, kg) significantly decreased after the intervention (baseline: 32.50 ± 8.57 kg, final: 29.66 ± 8.60 kg, ∆ = −2.84 ± 2.56, *p* < 0.001), as well as the muscle mass of the individuals (baseline: 30.15 ± 6.44 kg, final: 29.75 ± 6.46 kg, ∆ = −0.40 ± 0.77, *p* < 0.001).

Regarding the evaluated biochemical parameters, it was first observed that the baseline values of HOMA-IR (4.24 ± 3.27), HDL-C (36.30 ± 9.16 mg/dL), VLDL-C (32.66 ± 15.70 mg/dL), LDL-C (106.59 ± 26.78 mg/dL), and triglycerides (163.15 ± 78.49 mg/dL) were out of the normal range. The baseline insulin values (18.75 ± 12.46 µUI/mL) were slightly above the healthy range, and the baseline values of glucose (89.56 ± 11.46 mg/dL) and total cholesterol (175.55 ± 30.88 mg/dL) were considered normal.

After the intervention, significant and favorable changes were reported in the concentrations of almost all the evaluated biochemical parameters, except for HDL-c (∆ = −0.577 ± 5.42, *p* = 0.274), which remained unchanged throughout the intervention.

Glucose and insulin levels were significantly reduced (∆ = −3.65 ± 9.10, *p* < 0.001; −4.61 ± 9.83, *p* < 0.001, respectively), contributing to the reduction in HOMA-IR (∆ = −1.16 ± 2.39, *p* < 0.001). Total cholesterol concentrations showed significant improvement (∆ = −15.53 ± 20.48, *p* < 0.001), and it is noteworthy that the values of VLDL-c (27.82 ± 13.79 mg/dL, ∆ = −4.84 ± 12.76, *p* < 0.001), LDL-c (96.39 ± 23.93 mg/dL, ∆ = −10.19 ± 19.33, *p* < 0.001), and triglycerides (139.19 ± 68.91 mg/dL, ∆ = −23.96 ± 63.86, *p* < 0.001) decreased to within the normal range.

### 3.4. Interactions Between CETP rs708272 Genotypes and Baseline Trans-Fatty Acid Intake on Glucose, Insulin, and HOMA-IR

**Figure 1:**
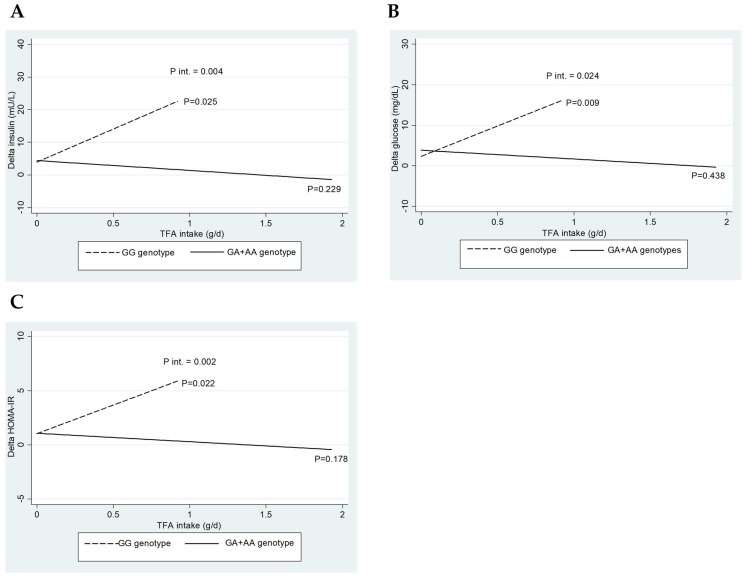
Impact of *CETP* rs708272 genotypes on the interactions of baseline trans-fatty acid intake on glucose, insulin, and HOMA index. (**A**) Interaction between *CETP* rs708272 genotypes and baseline trans-fatty acid intake concerning the change (∆) in insulin. (**B**) Interaction between *CETP* rs708272 genotypes and baseline trans-fatty acid intake concerning the ∆ in glucose. (**C**) Interaction between *CETP* rs708272 genotypes and baseline trans-fatty acid intake concerning the ∆ HOMA-IR. The interactions were adjusted for age, sex, BMI, and total energy intake.

Figure 1 depicts the interaction between *CETP* rs708272 genotypes (GG vs. GA+AA) and baseline TFA intake regarding the change (∆) in insulin (A), glucose (B), and HOMA-IR (C). Interestingly, only significant interactions were detected when consuming trans-fatty acids.

The three interaction graphs presented in Figure 1 show a similar phenomenon, as a significant interaction was observed between genotype and TFA intake on insulin levels (*p*-int = 0.004), glucose (*p*-int = 0.024), and HOMA-IR (*p*-int = 0.002). Similarly, lower reductions in insulin (*p* = 0.025), glucose (*p* = 0.009), and HOMA-IR (*p* = 0.022) were observed for the GG genotype with increasing TFA intake, whose effect was not observed for the GA+AA genotypes. This result indicates that the consumption of TFA significantly influences the reduction in the parameters, as mentioned above, only in carriers of the GG genotype of *CETP* rs708272.

## 4. Discussion

The current study aimed to investigate the interaction between the Cholesteryl Ester Transfer Protein (*CETP)* rs708272 polymorphism and the fatty acid intake on HOMA-IR indicators in overweight and obese adults following a hypocaloric diet for weight reduction. To the best of our knowledge, this is the first study to explore this interaction in the Mexican population, and the findings obtained were relevant and, furthermore, appropriate for the application of precision nutrition in the management of glucose metabolism markers.

The genotypic frequency of this study’s population resembles that Vargas-Alarcón et al. [22] reported in Mexican Mestizo individuals. Additionally, it is recognized that the population’s average age consists of young adults, aligning with findings by Occa et al. [23], who noted a higher willingness among this age group to participate in clinical trials. According to the nutrient intake evaluation, the intervention led to meaningful reductions in several critical nutritional areas, particularly in calories, carbohydrates, total lipids, saturated fats, and cholesterol, which could have positive health implications. Baseline data revealed that participants followed the Western diet pattern, characterized by high consumption of pre-packaged foods, refined grains, processed meat, high-sugar drinks, sweets, fried foods, high-fat dairy products, and high-fructose products. This dietary profile is primarily associated with a significant intake of simple carbohydrates, saturated fatty acids, cholesterol, and trans fatty acids (TFA) [24].

The healthy dietary pattern followed by the participants promoted improved dietary habits, reflected in the final nutritional evaluation. However, the consumption of TFA remained constant during the intervention. This result may be due to participants consuming processed foods and snacks with high TFA content, such as baked goods, pastries, and fried foods. Although public measures such as food labeling and taxation on unhealthy foods have been implemented in Mexico to encourage healthier choices [25], proactive steps are still required to address these issues and improve the nation’s overall health.

Abnormal anthropometric and body composition markers have been widely reported to be associated with metabolic disorders, such as hyperglycemia and insulin resistance, even in children [26,27]. Therefore, it is crucial to maintain these markers within healthy ranges to avoid progression to diabetes or other chronic non-communicable diseases.

After following the weight control diet, significant improvements were observed in this study’s anthropometric parameters. The caloric deficit imposed on individuals, along with the redistribution of macronutrient intake, contributed to these substantial reductions [28].

According to the World Health Organization [29], adopting a lifestyle that emphasizes healthy eating—such as increased consumption of fruits and vegetables, whole grains, and reduced processed foods—and enhanced physical activity constitute primary prevention strategies to mitigate the risk of non-communicable diseases. Such changes can potentially prevent up to 80% of cardiovascular diseases.

The provided nutritional plan in this trial effectively improved the evaluated biochemical parameters. It was interesting to observe that the population could be diagnosed with hypoalphalipoproteinemia according to the basal and final HDL-C values they presented. This finding is related to the fact that hypoalphalipoproteinemia is the most prevalent dyslipidemia in the Mexican population, followed by hypertriglyceridemia, which is partly due to the high prevalence of polymorphisms in the CETP, APOA1, and ABCA1 genes [30,31]. It is also essential to highlight that the presence of the G allele in several populations has been associated with altered CETP protein activity, resulting in lower HDL-C levels, which may increase the risk of cardiovascular diseases [22]. Another possible hypothesis proposes that obesity may be linked to an elevated rate of HDL catabolism, with excess adipose tissue contributing to the accelerated breakdown of both HDL and LDL [32].

In this context, it is well-established that overweight and obesity lead to higher plasma triglyceride (TG) levels. However, when a calorie-restricted diet is followed, TG levels typically decrease, primarily because of reduced VLDL-TG production due to lower substrate availability and decreased circulating insulin levels [33]. The decrease in HOMA-IR indicates improved insulin sensitivity, which is linked to lower fasting insulin levels. This finding aligns with existing research that shows similar results when caloric intake, refined carbohydrates, and saturated fats are regulated [34].

Precision Nutrition offers more precise and concise strategies, which can potentially increase the effect nutrition exerts on the human organism, either to prevent or as part of a comprehensive treatment to control diseases. Individuals possess distinct genetic variants that may affect their health unless dietary adjustments are made [35]. In this sense, one of the most important findings obtained in the present research indicates that the consumption of TFA significantly influences glucose, insulin, and HOMA-IR reductions in carriers of the GG genotype of *CETP* rs708272 SNP after dietary counseling. However, this effect was not observed in carriers of the AA or GA genotypes. Therefore, it is crucial to monitor the intake of TFA, especially in GG carriers, to control the glucose-related parameters mentioned above, ensure a successful eating plan, and prevent the potential progression of diabetes and cardiometabolic disease in these patients. However, in addition to TFA, the modulatory effect of other variables on glucose homeostasis cannot be discarded, including body weight loss, blood lipid features, caloric intake, and distribution of macro and micronutrients, as well as interactions with genetics.

Indeed, the pathophysiology of insulin resistance results from the interaction of various environmental and genetic factors that lead to dysregulation within the cellular environment [11]. To date, scientific literature has not described a specific mechanism of action that clarifies the effect of TFA intake on insulin resistance in carriers of the GG genotype of the SNP 708272 of the *CETP* gene. Nevertheless, evidence indicates that extracellular factors such as chronic subclinical inflammation, cellular hypoxia, lipotoxicity, and immunological abnormalities can cause intracellular stress in critical metabolic tissues, affecting normal insulin function and contributing to the development of insulin resistance [11,36].

**Figure 2:**
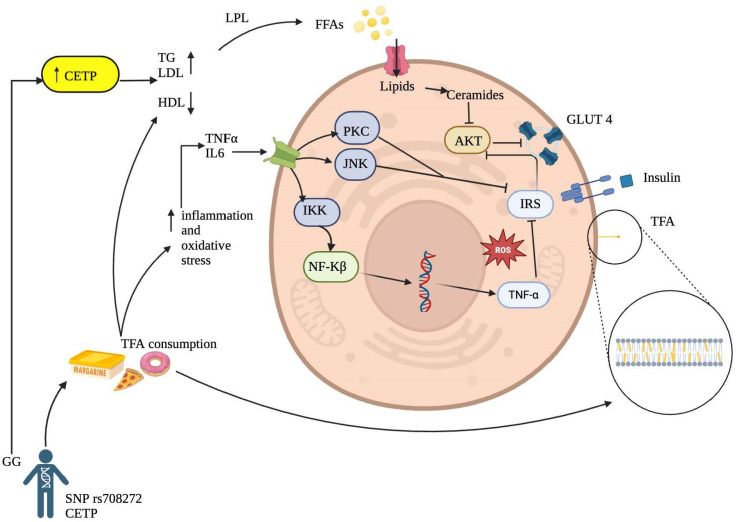
Proposed Mechanisms Linking Trans Fatty Acid Consumption to the Development of Insulin Resistance.

Although the precise mechanism of TFA on insulin resistance remains not fully understood, there are several molecular mechanisms that could explain this interaction (Figure 2).

Inflammatory Activation Pathway: It has been reported that TFA can increase inflammatory markers such as tumor necrosis factor-alpha (TNF-α) and interleukin-6, initiating a proinflammatory process in hepatocytes, adipocytes, myocytes, and other cell groups and tissues [37,38]. This process may stimulate major intracellular inflammatory pathways through increased expression of inflammatory factors involved in insulin resistance, such as protein kinase C (PKC), c-Jun N-terminal kinase (JNK), and the IκB kinase (IKK) complex that activates nuclear factor kappa B (NF-κB) in the cytoplasm. The final product of its transcription in the cell nucleus will produce more proinflammatory cytokines, ultimately inhibiting tyrosine phosphorylation of the insulin receptor substrate (IRS), thereby increasing insulin resistance [11,39]. This finding aligns with Castro and collaborators [36], who reported that TFA might affect essential fatty acid metabolism, prostaglandin balance, and endothelial function, increasing systemic inflammation by likely incorporating into the membranes of endothelial cells, monocytes/macrophages and adipocytes directly affecting inflammation-related signaling pathways.

Cell Membrane Alteration by Trans Fatty Acids: Another possible mechanism leading to insulin resistance associated with TFA intake is their ability to incorporate into cell membranes (Figure 2), primarily in phospholipids, altering physical properties such as membrane fluidity, resulting in a more rigid configuration due to the trans bonds of these fatty acids [40]. Likewise, consistent with Ballesteros and collaborators [37], the results suggest that TFA may affect carbohydrate metabolism after absorption by altering membrane lipids. This finding can, in turn, affect insulin sensitivity by modifying its interaction with the receptor and promoting membrane dysfunction in enzyme coupling.

It is crucial to emphasize the importance of controlling and monitoring TFA intake to prevent metabolic complications, particularly in individuals with the homozygous wild-type CETP rs708272 genotype, who underwent no reductions in glucose metabolism markers as increased TFA intake despite hypocaloric dietary intervention. Some strengths of this study include the implementation of a robust multivariable linear regression model using adjusted data, as well as the novelty of the results, which offer potential recommendations in the field of biomedical research and applied nutrition. As for the limitations, it is noteworthy that the findings pertain to populations from the western region of Mexico, with particular genetic and environmental backgrounds. However, this encourages studying other ethnically different populations and interactions with other lifestyle factors such as physical activity or sleep habits. Furthermore, the analysis of other hypocaloric diets with different macronutrient distributions and longer follow-up times is warranted.

## 5. Conclusions

CETP rs708272 GG carriers are more adversely affected by even a minimum TFA intake, experiencing significant persistence in no reducing glucose levels and insulin resistance parameters after dietary intervention. In contrast, individuals with GA+AA genotypes seem to exhibit a more stable metabolic response to increased TFA intake. These findings highlight the importance of considering genetic variants when evaluating dietary impacts on metabolic health.

## Figures and Tables

**Table 1:** Sociodemographic Characteristics and *CETP* rs708272 Genotypes.

Parameter	Total Population	Genotype (GG)	Genotype (GA+AA)	*p*
N=	78	24 (31%)	54 (69%)	-
Age	34.6 ± 8.1	36.0 ± 7.9	33.9 ± 8.2	0.28 *
Men	38 (48%)	14 (58%)	24 (44%)	0.37 ^#^
Women	40 (52%)	10 (42%)	30 (56%)	

Age in years is presented as mean ± standard deviation. Sex is presented as total and percentage. * Mann–Whitney Rank Sum Test. ^#^ χ^2^ test.

**Table 2:** Nutritional evaluation of the study population.

Component	Baseline	Final	∆	*p*
Energy (kcal)	1975.15 ± 663.78	1582.82 ± 580.43	−392.32± 833.04	<0.001 *
Protein (g)	98.0 ± 49.30	96.08 ± 25.84	−1.92 ± 54.05	0.941
Carbohydrates (g)	251.62 ± 164.12	177.78 ± 65.20	−73.83 ± 166.22	<0.001 *
Lipids (g)	78.73 ± 37.18	58.88 ± 24.22	−19.84 ± 45.18	<0.001 *
SFA (g)	24.63 ± 12.12	17.76 ± 9.32	−6.86 ± 15.59	<0.001 *
TFA (g)	0.3228 ± 0.4782	0.1069 ± 0.536	−0.106 ± 0.536	0.733
PUFA (g)	15.54 ± 11.23	12.33 ± 5.33	−3.212 ± 12.49	0.149
MUFA (g)	24.68 ± 14.98	20.51 ± 9.70	−4.17 ± 18.31	0.053
Cholesterol (mg)	397.258 ± 211.93	337.58 ± 165.27	−59.674 ± 216.99	0.020 *

SFA: Saturated fatty acids; TFA: Trans Fatty Acids; PUFA: Polyunsaturated fatty acids; MUFA: Monounsaturated fatty acids. Data presented as mean ± standard deviation; nutrient intake obtained from 24-h recall (baseline) and 3-day food diary (final); *: significant *p*-value < 0.05; ∆: final value—baseline value. The *p*-value ∆ was determined using the Wilcoxon test.

**Table 3:** Changes in anthropometric and biochemical parameters of participants.

Parameter	Baseline	Final	∆	*p*
Anthropometric parameters				
Body weight (kg)	86.35 ±15.13	82.86 ± 14.60	−3.48 ± 3.06	<0.001 *
BMI (kg/m^2^)	30.60 ± 4.08	29.34± 4.08	−1.26 ± 1.02	<0.001 *
Waist circumference (cm)	94.96 ± 12.14	90.85 ± 11.37	−4.10 ± 4.46	<0.001 *
Hip circumference (cm)	110.30 ± 7.91	107.31± 7.87	−2.98 ± 2.81	<0.001 *
Total body fat mass (kg)	32.50 ± 8.57	29.66 ± 8.60	−2.84 ± 2.56	<0.001 *
Muscle mass (kg)	30.15 ± 6.44	29.75 ± 6.46	−0.40 ± 0.77	<0.001 *
Biochemical parameters				
Glucose (mg/dL)	89.56 ± 11.46	85.91 ± 12.42	−3.65 ± 9.10	<0.001 *
Insulin (µUI/mL)	18.75 ± 12.46	14.14 ± 9.24	−4.61 ± 9.83	<0.001 *
HOMA-IR	4.24 ± 3.27	3.07 ± 2.32	−1.16 ± 2.39	<0.001 *
Total cholesterol (mg/dL)	175.55 ± 30.88	160.01 ± 29.31	−15.53 ± 20.48	<0.001 *
HDL-cholesterol (mg/dL)	36.30 ± 9.16	35.73 ± 8.80	−0.577 ± 5.42	0.274
VLDL-cholesterol (mg/dL)	32.66 ± 15.70	27.82 ± 13.79	−4.84 ± 12.76	<0.001 *
LDL cholesterol (mg/dL)	106.59± 26.78	96.39 ± 23.93	−10.19 ± 19.33	<0.001 *
Triglycerides (mg/dL)	163.15 ± 78.49	139.19 ± 68.91	−23.96 ± 63.86	<0.001 *

HDL: High-density lipoprotein, VLDL: very low-density lipoprotein, LDL: low-density lipoprotein; data presented as mean ± standard deviation; *: significant *p*-value < 0.05; ∆: final value—baseline value. The *p*-value ∆ was determined using the Wilcoxon test.

## Data Availability

The data presented in this study are available on request from the corresponding author. They are not publicly available for privacy reasons and because they are part of an ongoing study.
